# Effect of apolipoprotein A1 genetic polymorphisms on lipid profiles and the risk of coronary artery disease

**DOI:** 10.1186/s13000-015-0328-7

**Published:** 2015-07-16

**Authors:** BiHong Liao, KeQi Cheng, ShaoHong Dong, HuaDong Liu, ZhengLei Xu

**Affiliations:** Department of Cardiology, Second Clinical Medical College of Jinan University, Shenzhen People’s Hospital, Shenzhen, 518000 Guangdong Province China; Department of Internal Medicine, Second Clinical Medical College of Jinan University, Shenzhen People’s Hospital, NO. 1017 East Gate Road, Shenzhen, 518000 Guangdong Province China

**Keywords:** Apolipoprotein A1, Gene polymorphism, Coronary artery disease, Lipid

## Abstract

**Background:**

The disorder of lipid metabolism and genetic predisposition are major risk factors for coronary artery disease (CAD). Variants in the apolipoprotein A1 (*APOA1*) gene play an important role in the regulation of lipids. The objective of the present study was to investigate the effect of two polymorphisms (-75 G/A and +83 C/T) of *APOA1* on lipid profiles and the risk of CAD.

**Methods:**

A total number of 300 subjects with CAD and 300 age and sex matched healthy controls were enrolled for the study. Genotyping of the *APOA1* was performed by polymerase chain reaction-restriction fragment length polymorphism (PCR-RFLP) combined with gel electrophoresis, and then confirmed by direct sequencing.

**Results:**

The frequencies of *APOA1* -75 AA genotype [odds ratio (OR) =0.50, 95 % confidence interval (CI) = 0.28, 0.88; *P* = 0.02] and *APOA1* -75 A allele (OR =0.76, 95 % CI = 0.59, 0.98; *P* = 0.04) were significantly lower in CAD than in controls. The *APOA1* -75 A allele was significantly associated with increasing serum concentrations of ApoA1 and high-density lipoprotein cholesterol (HDL-C) (*P* < 0.001).

**Conclusions:**

The individuals with the *APOA1* -75 A allele were likely to have a lower risk of CAD as a result of its effect on higher serum concentrations of ApoA1 and HDL-C.

## Background

Coronary artery disease (CAD) is the leading cause of mortality and morbidity in the world [1]. Although the precise mechanisms responsible for the onset of CAD are still unknown, it is a multifactorial disease, which is influenced by interacting endogenous and exogenous factors [2, 3]. The major risk factors of CAD are age, stress, hypertension, diabetes mellitus, hyperlipidemia, smoking, high alcohol consumption, family history of CAD and obesity [4–8]. The disorder of lipid metabolism and genetic predisposition are major risk factors for CAD [9–11]. It has been estimated that genetic risk factors explain approximately 20 %-60 % of CAD cases [12]. Some genes have been investigated for their involvement in the development of CAD [13, 14].

Apolipoprotein A1 (apoA1) is the major apolipoprotein constituent of the high-density lipoprotein (HDL) and is involved in reverse cholesterol transport [15]. Up to now, several single-nucleotide polymorphisms (SNPs) have been identified in the *APOA1* gene located on the long arm of chromosome 11 [16]. A common G-to-A transition located -75 base pairs (bp) upstream from the transcription start site of the *APOA1* gene has been studied extensively [17]. Another polymorphism site was identified in the first intron of the *APOA1* gene that involves transition of C-to-T at +83 bp that has also been shown to affect HDL and apoA1 levels [18, 19].

Although three case-control studies have investigated the association between *APOA1* polymorphisms (-75 G/A and +83 C/T) and the risk of CAD, these studies have reported conflicting results [20–22]. The objective of the present study was to investigate the effect of two polymorphisms (-75 G/A and +83 C/T) of *APOA1* on lipid profiles and the risk of CAD in a Chinese population.

## Methods

### Study population

A total number of 300 subjects with CAD and 300 age and sex matched healthy controls were enrolled for the study between July 2011 and June 2014 in the Second Clinical Medical College of Jinan University, China. CAD cases were defined as those having severe angiostenosis (>50 %) in at least one major coronary artery determined by angiography based on WHO criteria. CAD cases were selected randomly from patients admitted to the hospital and who fulfilled the inclusion criteria. The control group was composed of age and sex matched healthy subjects who had undergone a coronary angiography in the same recruitment period as the CAD patients, with normal ECG, negative stress test, without family history of CAD or other cardiovascular diseases or angiographic evidence of CAD. The study was approved by the Institutional Ethical Committee of the Second Clinical Medical College of Jinan University. A signed informed consent was obtained from all participants in the study. Venous blood was collected in evacuated tubes after an overnight fast of 12 to 14 h.

### Biochemical analysis

Serum total cholesterol (TC), triglycerides (TG), high-density lipoprotein cholesterol (HDL-C) were measured by the clinical chemistry department using commercial kits following the manufacturer instructions. Serum low-density lipoprotein cholesterol (LDL-C) and very-low-density lipoprotein cholesterol (VLDL-C) were calculated using the Friedwald’s formula. The immunoturbidimetric assay was used to quantify the plasma concentrations of ApoA1 and ApoB.

### DNA extraction and genotyping

Total genomic DNA was isolated from peripheral blood leukocytes by the commercially available Qiagen kit (QIAGEN Inc., Valencia, CA, USA). Polymerase chain reaction restriction fragment length polymorphism (PCR-RFLP) assay was performed to assess the *APOA1* gene polymorphisms. Based on the GenBank reference sequence, the PCR primer pair used as follows: forward: 5ʹ-AGG GAC AGA GCT GAT CCT TGA ACT CTT AAG-3ʹ, and reverse: 5ʹ-TTA GGG GAC ACC TAG CCC TCA GGA AGA GCA-3ʹ. The amplified PCR products were digested with 10 units of the restriction endonuclease enzyme *Msp*I overnight. The digested fragments were resolved on a 3 % agarose gel and stained with ethidium bromide for visualization under UV light. The presence of the *Msp*I restriction stie at -75 bp (G allele) and at +83 bp (C allele) in the 433 bp product resulted in four fragments of 45, 66, 113 and 209 bp. The absence of the restriction site at -75 bp (A allele) resulted in three fragments of 45, 179 and 209 bp. The absence of the restriction site at +83 bp (T allele) created a larger fragment of 254 bp instead of two fragments of 45 and 209 bp. For quality control, we performed double sampling PCR-RFLP in more than 10 % of the samples and found no differences, and then confirmed by direct sequencing.

### Statistical analysis

Statistical analyses were performed by SPSS software for Windows (SPSS 11.0 SPSS Inc., Chicago, IL). Allele frequencies were calculated by the gene counting method. Differences between continuous variables were assessed by Student’s *t* test, while those between categorical variables were evaluated using *x*^2^ test. The existence of differences in genotypic frequencies between groups was assessed by means of *x*^2^ test. The odds ratio (OR) and 95 % confidence intervals (CI) were also calculated. *P* < 0.05 was required for statistical significance.

## Results

### Characteristics of participants

Distribution of the various phenotypic variables in cases and controls were shown in Table 1. No significant differences were found between the CAD cases and controls in age, sex, hypertension and ApoB (Table 1). HDL-C and ApoA1 were significantly decreased (*P* < 0.001), but TG (*P* < 0.001), TC (*P* = 0.003), LDL-C (*P* < 0.001) and VLDL-C (*P* = 0.004) levels were significantly increased in patients as compared to controls. Multiple logistic regression analysis of known predictors of CAD confirmed the independent role of smoking status (*P* < 0.001), diabetes (*P* = 0.001), obesity (*P* = 0.001), hyperlipidemia (*P* < 0.001) and ApoA1 (*P* < 0.001) as risk factors of CAD (Table 1).

**Table 1:** Distribution of the various phenotypic variables in cases and controls

	CAD (*n* = 300)	Controls (*n* = 300)	*P*
Age (years)	56.7 ± 9.9	56.1 ± 9.7	0.45
Sex (Male/Female)	238/62	231/69	0.49
Smoking status (Ever/Never)	84/216	46/254	<0.001
Diabetes (Positive/Negative)	74/226	42/258	0.001
Hypertension (Positive/Negative)	163/137	144/156	0.12
Obesity (Positive/Negative)	87/213	51/249	0.001
Hyperlipidemia (Positive/Negative)	93/207	49/251	<0.001
TG (mg/dL)	192.1 ± 86.7	167.5 ± 77.4	<0.001
TC (mg/dL)	198.7 ± 58.4	185.3 ± 50.1	0.003
HDL-C (mg/dL)	33.7 ± 11.6	38.9 ± 12.9	<0.001
LDL-C (mg/dL)	126.6 ± 37.4	112.9 ± 33.8	<0.001
VLDL-C (mg/dL)	38.4 ± 21.5	33.5 ± 20.3	0.004
ApoA1 (mg/dL)	123.5 ± 22.3	131.9 ± 23.5	<0.001
ApoB (mg/dL)	91.4 ± 27.3	90.6 ± 26.7	0.72

Abbreviations: CAD, coronary artery disease; TG, triglyceride; TC, total cholesterol; HDL-C, high-density lipoprotein cholesterol; LDL-C, low-density lipoprotein cholesterol; VLDL-C, very-low-density lipoprotein cholesterol; Apo, apolipoprotein

### APOA1 -75 G/A polymorphisms, lipids and CAD

The frequencies of *APOA1* -75 AA genotype (OR = 0.50, 95 % CI = 0.28, 0.88; *P* = 0.02) and *APOA1* -75 A allele (OR =0.76, 95 % CI = 0.59, 0.98; *P* = 0.04) were significantly lower in CAD than in controls (Table 2). The *APOA1* -75 A allele was significantly associated with increasing serum concentrations of ApoA1 and high-density lipoprotein cholesterol (HDL-C) (*P* < 0.001) (Table 3).

**Table 2:** Genotype and allele frequencies of APOA1 gene polymorphisms (-75 G/A and +83 C/T) among CAD cases and healthy controls

	Cases (*n* = 300)	Controls (*n* = 300)	OR (95%CI)	*P*
Genotype				
GG	175(58.3)	161(53.7)	1.00(Reference)	
GA	104(34.7)	100(33.3)	0.96(0.68,1.36)	0.80
AA	21(7.0)	39(13.0)	0.50(0.28,0.88)	0.02
CC	221(73.7)	230(76.7)	1.00(Reference)	
CT	54(18.0)	39(13.0)	1.44(0.92,2.26)	0.11
TT	25(8.3)	31(10.3)	0.84(0.48,1.47)	0.54
Allele				
G	454(75.7)	422(70.3)	1.00(Reference)	
A	146(24.3)	178(29.7)	0.76(0.59,0.98)	0.04
C	496(82.7)	499(83.2)	1.00(Reference)	
T	104(17.3)	101(16.8)	1.04(0.77,1.40)	0.82

APOA1, apolipoprotein A1; CAD, coronary artery disease; OR, odds ratio; CI, confidence interval

**Table 3:** Lipid profiles of CAD cases and controls according to APOA1 -75 G/A polymorphisms

	CAD			*P* value	Controls			*P* value
	GG	GA	AA		GG	GA	AA	
TG (mg/dL)	187.1 ± 79.3	198.9 ± 92.5	175.5 ± 86.2	0.72	160.5 ± 57.3	176.1 ± 62.4	156.9 ± 59.8	0.68
TC (mg/dL)	192.4 ± 55.7	206.4 ± 59.4	203.3 ± 56.8	0.44	178.9 ± 48.7	194.8 ± 53.6	191.4 ± 50.2	0.39
HDL-C (mg/dL)	30.4 ± 10.6	36.8 ± 11.9	41.5 ± 12.7	<0.001	37.1 ± 12.0	41.2 ± 13.6	46.8 ± 14.5	<0.001
LDL-C (mg/dL)	124.6 ± 36.9	129.8 ± 40.1	126.7 ± 38.2	0.87	109.7 ± 30.5	118.4 ± 36.5	113.2 ± 33.7	0.69
VLDL-C (mg/dL)	37.4 ± 12.4	39.8 ± 19.7	35.1 ± 10.6	0.31	32.1 ± 17.6	35.2 ± 18.4	31.4 ± 16.2	0.42
ApoA1 (mg/dL)	114.5 ± 20.7	127.6 ± 22.5	138.9 ± 24.1	<0.001	124.4 ± 23.5	135.7 ± 24.3	144.7 ± 25.8	<0.001
ApoB (mg/dL)	86.7 ± 25.4	97.6 ± 29.5	90.7 ± 26.9	0.63	88.1 ± 25.8	93.6 ± 27.5	89.5 ± 26.7	0.74

CAD, coronary artery disease; APOA1, apolipoprotein A1; OR, odds ratio; CI, confidence interval; TG, triglyceride; TC, total cholesterol; HDL-C, high-density lipoprotein cholesterol; LDL-C, low-density lipoprotein cholesterol; VLDL-C, very-low-density lipoprotein cholesterol; Apo, apolipoprotein

### APOA1 + 83 C/T polymorphisms, lipids and CAD

No association was found between *APOA1* + 83 C/T polymorphisms and risk of CAD (Table 2).

## Discussion

Many studies have been conducted to test the association of SNPs and CAD. A meta-analysis of thirteen case-control studies suggested that thrombomodulin -33G/A and Ala455Val polymorphisms were risk factors for CAD [23, 24]. A meta-analysis of 72 studies including 23,557 cases and 21,526 controls suggested that plasminogen activator inhibitor-1 4G/5G polymorphism was a risk factor for CAD [25]. A meta-analysis of 10,617 cases and 8,302 controls from 37 studies revealed that endothelial nitric oxide synthase 4b/a polymorphisms could be a risk factor for developing CAD, particularly in African populations and population-based subgroups [26]. A meta-analysis of 21 eligible literatures confirmed a protective effect of C242T polymorphism of CYBA gene on CAD in Asian population and indicated that A640G polymorphism of CYBA gene was significantly associated with decreased risk of CAD [27]. A meta-analysis of ten studies suggested there was an increase in the risk of CAD conferred by the E-selectin Ser128Arg polymorphism and the P-selectin thr715Pro polymorphism may be a protective factor of myocardial infarction [28]. A meta-analysis of eighteen studies with 3,546 cases and 3,852 controls suggested that the intercellular adhesion molecule-1 K469E polymorphism was a risk factor for CAD [29]. A meta-analysis of thirty case-control studies suggested that EcoRI and SpIns/Del polymorphisms of the APO B gene significantly increased the risk of CAD [30].

The *APOA1* gene polymorphisms were also extensively studied and reported to be associated with other diseases. A hospital-based case-control study found that *APOA1* -75 AA genotype was associated with a higher acute lung injury (ALI) risk after cardiopulmonary bypass (CPB) surgery. Those patients with the *APOA1* -75 AA genotype and A allele had higher 30-day mortality of ALI after CPB surgery [31]. A case-control study found an association of the *APOA1* -75G/A promoter polymorphism with cognitive performance in multiple sclerosis [32]. A prospective case-control study found a positive association between *APOA1* -75 A allele carriers and breast cancer risk [33]. A pilot study in a north Indian population suggested that *APOA1* polymorphisms (-75 G/A and +83 C/T) might be susceptibility to myocardial infarction [19]. A case-control study found the *APOA1* -75 A allele was associated with an increased risk for Alzheimer's disease [34]. A Brazilian elderly cohort showed that *APOA1* polymorphisms (-75 G/A and +83 C/T) could be as risk factors for hypertension and obesity [35]. A case-control study suggested that the *APOA1* -75 G/A polymorphism was associated with gallstone disease and shows sex-specific differences [36].

Although our results revealed that the individuals with the *APOA1* -75 A allele were likely to have a lower risk of CAD as a result of its effect on higher serum concentrations of ApoA1 and HDL-C, the exact mechanism is still unclear. However, the results have been inconsistent and inconclusive, with few studies reporting either no association or negative association between *APOA1* -75 A allele and plasma lipids [37–39]. Our study was consistent with some other studies [17, 40–44].

Several shortcomings of the present study should be discussed. Firstly, CAD, a multifactorial disease, is influenced by interacting endogenous and exogenous factors, which were not explored in the present study. Secondly, the subjects in this research are only from Han Chinese ethnic group. It would be interesting to conduct similar studies in different populations for comparison. Thirdly, about 50 % cases took statins by which lipid levels are affected. We have adjusted for medicine use in this analysis, but no information could be received on the baseline lipid levels of these cases. Finally, the potential selection bias cannot be avoidable, because this is a hospital based case control study and the subjects may not be representative of the general population.

## Conclusions

In conclusion, our results indicated that the individuals with the *APOA1* -75 A allele were likely to have a lower risk of CAD as a result of its effect on higher serum concentrations of ApoA1 and HDL-C. Additional studies are needed to confirm this finding.
